# Pollinators do not avoid or prefer a locally rare pollen colour morph in *Erythronium americanum* (Liliaceae)

**DOI:** 10.17912/micropub.biology.001286

**Published:** 2025-03-04

**Authors:** Jada A. Ripley, Gwenyth A. Deadman-Wylie, Amanda L. Traylor, Emily J. Austen

**Affiliations:** 1 Biology Department, Mount Allison University, Sackville, NB, Canada

## Abstract

The red-yellow pollen colour polymorphism that occurs in populations of *Erythronium americanum* provides an excellent opportunity to investigate the conditions leading to maintenance of phenotypic variation. Using arrays of cut *E. americanum* flowers with red- or yellow-pollen set in a naturally monomorphic red-pollen population, we tested whether pollinators exhibit frequency-dependent behaviours *.* As was previously observed when pollinator preference was tested in a pollen-colour polymorphic population, pollinators accustomed to the monomorphic red-pollen population in this study exhibited no pollen colour preference among flowers cut from a polymorphic population. The lack of pollinator colour preference against either a polymorphic or red-monomorphic background suggests that pollinators do not impose frequency-dependent selection. It is possible, however, that results would differ against a monomorphic yellow-pollen background.

**Figure 1. Pollinators exhibit no preference for red- or yellow-pollen morphs of Erythronium americanum against the backdrop of a monomorphic red-pollen population:**
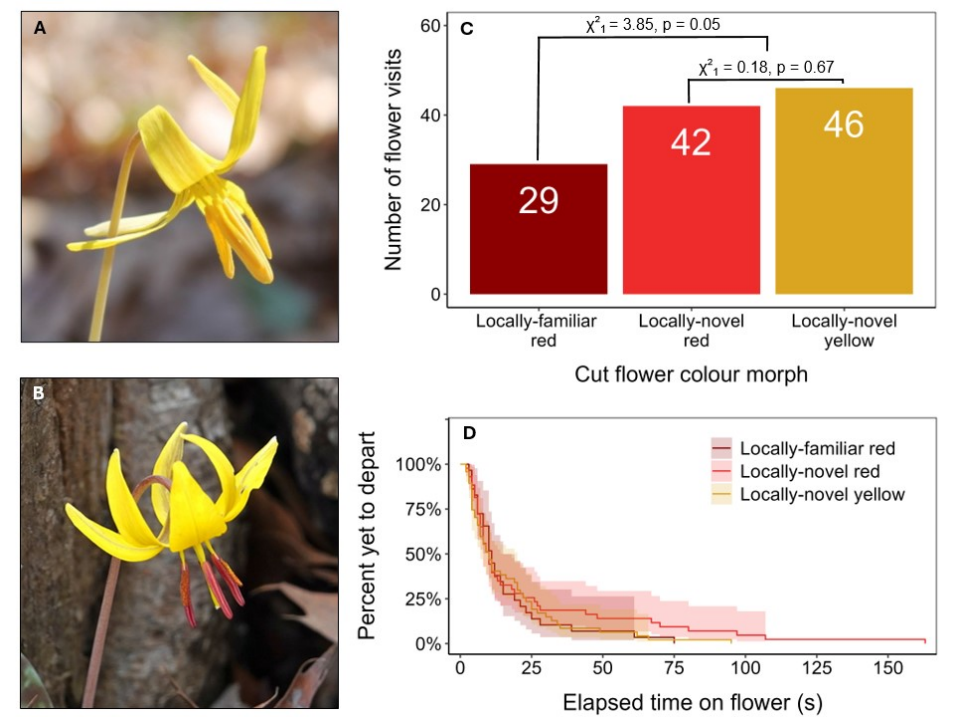
(A) The yellow-pollen morph (Emily Austen. CC BY-NC 4.0 2016) and (B) red-pollen morph (Ian Whyte. CC0 1.0 2024) frequently co-occur within *E. americanum* populations. (C) When offered arrays of cut flowers with red or yellow pollen from a polymorphic population, pollinators within a monomorphic red-pollen population visited the two morphs at random (χ2 _df = 1_ = 0.18182, p = 0.6698, >0.05). (D) Similarly, pollinators did not alter the duration of their flower visits depending on pollen morph (Kaplan-Meier survival curves, ribbons depict 95% CI).

## Description

By virtue of being readily detected and easily quantified, naturally occurring colour polymorphisms are an excellent tool for understanding the evolutionary processes that maintain phenotypic variation within populations (Svensson 2017) . In plants, petal colour polymorphisms have been a particular focus (Sapir et al. 2021) , providing examples of mutation-selection balance (e.g. *Delphinium nelsonii* , Waser & Price 1981), spatially- and temporally-variable selection (e.g., *Linanthus parryae* , Schemske & Bierzychudek 2001), and conflicting selection by different agents (e.g. *Claytonia virginica* , Frey 2004). Overall, colour polymorphisms provide the opportunity to define general conditions that tip the balance of drift, geneflow, selection, and mutation towards the maintenance or loss of variation.

Some plant species, including *Erythronium americanum* Ker Gawl (Liliaceae, a long-lived spring ephemeral of Eastern North America), exhibit pollen colour polymorphism, but not petal colour polymorphism. Individual *E. americanum* plants produce a single yellow flower bearing either yellow or red pollen ( Figure 1A and B), and an estimated >40% of *E. americanum* populations are pollen-colour polymorphic (Austen et al. 2018) . A previous study (Austen et al. 2018) found no clear mechanism by which pollen colour variation might be maintained within *E. americanum* populations: pollen colour did not affect fruit production following single-donor crosses, pollen tube growth following UV-B exposure, attraction of pollen-eating *Ischnomera ruficollis* Say beetles (formerly *Asclera ruficollis* , Oedemeridae), or attraction of most pollinator taxa. Experiments were, however, carried out in populations where both the red- and yellow-pollen morphs were common, preventing detection of any frequency-dependent effects.

Negative frequency-dependent selection, in which a rare morph has a fitness advantage over more common morphs, is a powerful form of balancing selection. Absent strong genetic drift, negative frequency-dependent selection is predicted to maintain morph diversity indefinitely. It is negative frequency-dependent selection, for example, that has allowed variants at self-incompatibility loci to persist through entire clades (Delph & Kelly 2014) . Although surprisingly uncommon as a force maintaining flower colour variation— a recent review found that just 1 of 37 papers supported negative frequency-dependent selection as a driving mechanism (Sapir et al. 2021: Table 1)— the possibility of negative frequency-dependent selection on pollen colour in *E. americanum* remains open.

We tested whether pollinators exhibit frequency-dependence in their visits to *E. americanum* flowers with red *vs.* yellow pollen by observing visitation to arrays of flowers cut from a polymorphic population against the backdrop of a monomorphic red-pollen population. Preference for the locally-novel yellow morph by putative pollinators would suggest that frequency-dependent behaviours by pollinators can help to maintain pollen colour variation in *E. americanum* populations. While frequency-dependence can be tested in some other systems by introducing arrays of varying morph frequency in areas where the study system does not occur (e.g., *Dactylorhiza sambucina* , Gigord et al. 2001), the scarcity of floral resources in the early-spring flowering season of *E. americanum* makes this approach infeasible for our study. Pollinator activity would likely be too infrequent away from the preferred floral resources.

In total, across 31 observation periods of approximately 20 minutes each (620 minutes total over a two-day period during peak flowering), we observed 77 putative pollinators interact with 117 flowers in the arrays. "Putative pollinator” includes any visitor ( *Bombus* , *Andrena,* other small bees, Bombyliidae *,* other unidentified flies *,* and one unidentified small beetle) that interacted with stamens/pistil, rested on petals, or foraged for nectar. These putative pollinators exhibited no preference for the red-pollen morph *vs.* yellow-pollen morph cut from the polymorphic population ( Figure 1C, χ2 _df = 1_ = 0.18182, p = 0.6698, >0.05). Pollinators tended, however, to prefer cut flowers from the polymorphic population over cut flowers from the local monomorphic population ( Figure 1C, χ2 _df = 1_ = 3.84615, p = 0.05). We suspect this preference arose because, for logistical reasons, the flowers from the polymorphic population were collected as buds the day prior and allowed to open in the lab, while those from the monomorphic population were collected as open flowers on the day of observation. Flowers from the polymorphic population had therefore not yet been visited when presented in the array, while the local flowers may have had some pollen already removed. Nonetheless, the lack of preference for one morph or the other from the polymorphic population is evidence that pollinators do not strongly prefer or avoid a familiar (in this case, red) pollen morph in this system.

Not only did putative pollinators not show any morph preference in visiting flowers, they also did not alter their visit duration by pollen morph. For example, the expected time at which 50% of visitors will have left a flower is approximately 10 seconds for all three groups ( Figure 1D ), and although red-pollen flowers from the polymorphic population had occasional long visits, the 95% CI for probability of departure at, for example, 60 seconds, are broadly overlapping across the three flower groups ( Figure 1D ). Results for both probability of visitation and visit duration are qualitatively unaffected when we reduce the dataset to 60 flower visits by 45 visitors that were specifically observed to be foraging for nectar or pollen. Our results are also qualitatively similar to those of a previous study of pollinator preferences against the background of polymorphic *E. americanum* populations (Austen et al. 2018) . We therefore do not expect any pollen transfer advantage (or disadvantage) for a rare morph in *E. americanum* .

Overall, our results point to an absence of frequency-dependence in pollinator visitation to red- and yellow-pollen morphs of *E. americanum* in this population. We note, however, frequency-dependent selection by pollinators in monomorphic yellow-pollen populations of *E. americanum* is yet to be investigated.

We did not, in either this work or in our past experiments, notice any latency in the ability of pollinators to discover flowers of either pollen morph. The large size of the anthers (~12 mm long when undehisced, EJA *pers. obs* .), and their projection from the flower surface with tepals strongly reflexed ( Figure 1A and B), suggests that they contribute to pollinator attraction to the flower. Previous work in this system suggests that the red-pollen morph may be more strongly discriminated from the yellow tepals of the flowers at a peak spectral sensitivity (~540 nm) of at least one pollinator ( *Bombus impatiens* ), while the yellow-pollen morph may be better distinguished from a background of dry leaf litter and mottled-green *E. americanum* leaves (Austen et al. 2018: Figure S2). Data on spectral sensitivities are unavailable for other pollinators of *E. americanum* .

The mechanism maintaining variation in pollen colour in this system remains unclear. Neutral processes have been implicated in maintaining flower colour polymorphism in *Iris lutescens* (Wang et al. 2016) and may be contributing to the maintenance of pollen colour variation in *E. americanum* . Another possibility that merits investigation is preference for a common morph by pollen-eating *Ischnomera ruficollis.* Due to their absence at our study site, we were unable to observe *I. ruficollis* preferences. However, preference for the locally-familiar red-pollen morph by antagonistic pollen-eating beetles would be consistent with frequency-dependent selection maintaining pollen colour variation. Finally, correlations of pollen colour with other traits through pleiotropy or linkage, possibly leading to conflicting selection through different fitness components, should also be examined (e.g. Twyford et al. 2018, Koski et al. 2020).

Pollen colour polymorphism is reported less frequently than petal colour polymorphism. Analogous variation in animal sperm seems to be similarly rare: we could find no reports of within-population sperm polymorphism (but see Swallow & Wilkinson 2002 and Briones et al. 2012 for examples of within-individual and between-population polymorphism, respectively). Case studies like *E. americanum* , its pollen-colour polymorphic congeners (Thomson 1986, Lin & Rausher 2024) , and other more distantly-related taxa including *Campanulastrum americanum* (Campanulaceae, Ison et al. 2019, Koski et al. 2020) and *Nigella digenii* (Ranunculaceae, Jorgensen and Andersson 2005) allow us to investigate conditions that maintain variation where it is not expected. To date, no evidence of negative frequency-dependent selection has been reported in any of these pollen-colour polymorphic systems.

## Methods

Observations were carried out during peak flowering (May 13 and 14, 2022) at a monomorphic red-pollen population of *E. americanum* (“Ogden Brook”, Table 1) in Sackville, NB, Canada. We created arrays of cut flowers with red- *vs.* yellow-pollen to observe pollinator and pollen-consumer preferences against the monomorphic red-pollen background. Red- and yellow-morph flowers were differentiated on site based on observable colour and contrast. We did not use quantitative analyses (e.g. with spectrometer) to compare pollen colours, and so it is possible that the red-pollen morph from the monomorphic population differed from the red-pollen morph from the polymorphic population. However, (1) even if such difference did occur, the difference between the two reds would be much less than that between the red and yellow morphs ( Figure 1 ), and (2) we think it is more likely that there is quantitative variation within the red morph at both sites, as described in Austen et al. 2018.

Arrays included three flowers each: one yellow-morph and red-morph each, cut from a polymorphic population ~19km away (“Dorchester Cape”, Table 1), and, to test for preference for local clones, one red-morph from the monomorphic Ogden Brook population. Unopened flower buds were collected from the polymorphic Dorchester Cape population in the afternoon, stored in water picks on a lab bench overnight, and brought to the field the following morning; flowers from the monomorphic Ogden Brook population were cut on the morning of observation. Flowers were displayed singly in water picks with *E. americanum* leaves, arranged in an 8cm triangle in a foam tray partially covered with leaf litter. Arrays were observed for 20 minutes at a time; a total of 31 observations were completed.

We narrated descriptions of each observed visit to a flower on voice recorders, noting the visitor’s arrival on a flower, a description of the visitor, the visitor’s behaviour, and its departure from the flower. From these recordings, we extracted data on the identity of the flower visited and the duration of visit. We used chi-square tests to analyze the frequency of visit to flowers with red- *vs.* yellow-pollen from the polymorphic population (expected frequency 1:1), and the frequency of visits to red-morph flowers from the monomorphic population *vs.* any morph from the polymorphic population (expected frequency 1:2). We analyzed duration of visit as a function of flower type (red-morph from monomorphic population, red-morph from polymorphic, or yellow-morph from polymorphic) in Kaplan-Meier survival analysis. A survival analysis framework is appropriate for event-time data, as occur in several ecological contexts (e.g., arrival of a migrant, abscission of a flower, duration of behavioural display: Muenchow 1986, or, in our case, duration of visit to a flower). Chi-square test statistics were calculated by hand; the Kaplan-Meier model was run and plotted using function survfit from R package survival (R Core Team 2022, Therneau 2022, Therneau & Grambsch 2000) within the R studio interface (RStudio Team 2022) .

## Reagents

**Table:** 

Species	Population	Population pollen colour	Latitude (ºN)	Longitude (ºW)
*Erythronium americanum*	Ogden Brook	Monomorphic red	45.93546	– 64.41689
*Erythronium americanum*	Dorchester Cape	Polymorphic red and yellow	45.84337	– 64.521961
